# Modulation of Adhesion Molecules Expression by Different Metalloproteases Isolated from *Bothrops* Snakes

**DOI:** 10.3390/toxins13110803

**Published:** 2021-11-15

**Authors:** Bianca C. Zychar, Patrícia B. Clissa, Eneas Carvalho, Adilson S. Alves, Cristiani Baldo, Eliana L. Faquim-Mauro, Luís Roberto C. Gonçalves

**Affiliations:** 1 Laboratory of Pathophysiology, Butantan Institute, São Paulo 05503-900, Brazil; 2 Laboratory of Immunopathology, Butantan Institute, São Paulo 05503-900, Brazil; patricia.clissa@butantan.gov.br (P.B.C.); eliana.faquim@butantan.gov.br (E.L.F.-M.); 3 Laboratory of Bacteriology, Butantan Institute, São Paulo 05503-900, Brazil; eneas.carvalho@butantan.gov.br; 4 Department. of Physiology and Biophysics, Institute of Biomedical Sciences, University of São Paulo, São Paulo 05503-900, Brazil; adilson@icb.usp.br; 5 Department of Biochemistry and Biotechnology, State University of Londrina, Paraná 86051-990, Brazil; critianibaldo@uel.br

**Keywords:** *Bothrops*, metalloproteases, inflammation, microcirculation, adhesion molecules, leukocyte-endothelium interactions

## Abstract

Snake venom metalloproteinases (SVMP) are involved in local inflammatory reactions observed after snakebites. Based on domain composition, they are classified as PI (pro-domain + proteolytic domain), PII (PI + disintegrin-like domains), or PIII (PII + cysteine-rich domains). Here, we studied the role of different SVMPs domains in inducing the expression of adhesion molecules at the microcirculation of the cremaster muscle of mice. We used Jararhagin (Jar)—a PIII SVMP with intense hemorrhagic activity, and Jar-C—a Jar devoid of the catalytic domain, with no hemorrhagic activity, both isolated from *B. jararaca* venom and BnP-1—a weakly hemorrhagic P1 SVMP from *B. neuwiedi* venom. Toxins (0.5 µg) or PBS (100 µL) were injected into the scrotum of mice, and 2, 4, or 24 h later, the protein and gene expression of CD54 and CD31 in the endothelium, and integrins (CD11a and CD11b), expressed in leukocytes were evaluated. Toxins induced significant increases in CD54, CD11a, and CD11b at the initial time and a time-related increase in CD31 expression. In conclusion, our results suggest that, despite differences in hemorrhagic activities and domain composition of the SVMPs used in this study, they behave similarly to the induction of expression of adhesion molecules that promote leukocyte recruitment.

## 1. Introduction

Snakebite accidents in humans are a severe global health problem that mainly affects the poor and economically active population, causing significant social issues [1,2]. The World Health Organization (WHO) estimates that approximately 2.7 million snakebite accidents occur annually worldwide. As a result of these accidents, an estimated 81,000 to 138,000 deaths occur, and 400,000 survivors experience permanent sequelae [3,4].

In Brazil, an average of 29,000 snakebites occur annually, resulting in approximately 120 deaths/year [5], in addition to underreported cases. Of these 29,000 envenomations, approximately 90% are caused by snakes of the *Bothrops* genus [6].

In *Bothrops* snakebites, severe tissue loss at the site of the bite is observed as a result of hemorrhage and an exacerbated local inflammatory response induced by the venom.

Among the components present in these venoms, metalloproteases are considered to be some of the main factors responsible for local inflammation and necrosis [7,8,9].

Some of the most abundant proteins found in *Bothrops* venoms are snake venom metalloproteinases (SVMPs), zinc-dependent proteinases that belong to the reprolysin subfamily [10]. Analysis of the gene expression in the venom gland of *Bothrops jararaca* (*Bj*) snakes showed that more than 50% of transcribed genes are SVMPs [10]. SVMPs are classified as PI to PIII, according to the presence or absence of disintegrin and cysteine-rich domains together with a typical metalloproteinase domain, at least in the precursor molecule form [11].

Several hemorrhagic metalloproteases have been isolated from *Bothrops jararaca* venom (*Bj*V) [12,13,14]. One of the best characterized proteins is jararhagin (Jar), a protein with a molecular weight of 52 kDa that contains PI, metalloproteinase, ECD-disintegrin (ECD: Glu-Cys-Asp) and cysteine-rich domains [14], which are characteristics of an SVMP of the PIII class. In general, the toxins belonging to this class are highly hemorrhagic, and this activity depends on the metalloproteinase domain. However, ECD-disintegrin and cysteine-rich domains have also been shown to be important for the biological functions of these toxins [15,16,17].

Two forms of Jar are present in *Bj*V: the molecule with the three domains, as described above, and jararhagin-C (Jar-C), a nonhemorrhagic molecule containing only the ECD-disintegrin and cysteine-rich domains produced by the proteolytic cleavage of Jar [18]. The absence of the catalytic site in this toxin does not prevent the inflammatory activity of the toxin. Jar-C triggers the local release of cytokines and induces alterations in leukocyte-endothelium interactions, similar to the inflammatory response caused by Jar [9,19].

PI-class SVMPs contain only a catalytic domain, have a molecular mass ranging from 20 to 30 kDa, possess fibrin(ogeno)lytic activity, and mostly present weak hemorrhagic activity. Metalloproteases isolated from *Bothrops neuwiedi* venoms (*Bn*V), such as neuwiedase (PI-class SVMP), degrade fibrinogen, fibrin, type I collagen, fibronectin, and laminin and induce inflammatory reactions [20].

Two other PI-SVMPs analogous to neuwiedase were isolated from *Bn*V: BnP1 and BnP2. However, only BnP1 exerts biological effects similar to those of Jar on the hydrolysis of coagulation factors, the degradation of extracellular matrix components, and the apoptosis induction in endothelial cells. Nevertheless, Jar possesses strong hemorrhagic activity, while BnP1 presents weak hemorrhagic activity [21].

The inflammatory response starts with sequential and orchestrated phenomena in the vascular endothelium, resulting in the loss of vasomotor reactivity and cell recruitment. Endothelial dysfunction causes an imbalance in microcirculatory homeostasis, activating the immune response that triggers the production and release of inflammatory mediators, such as tumor necrosis factor-alpha (TNF-α), interleukin-1 (IL-1) and interleukin-6 (IL-6) and the subsequent release of vasoactive substances, alteration of blood flow and increased vascular permeability and leukocyte migration to inflamed tissues after *Bothrops* envenomation [19,22].

In addition, the release of these chemotactic agents mediates a sequence of adhesive contacts between leukocytes and endothelial cells [23]. These adhesive contacts, as well as the exit of these cells from postcapillary venules, are mediated by the expression of adhesion molecules [24]. Leukocyte transmigration to tissue is composed of a complex series of events depending on the time and expression levels of various adhesion molecules through a precise mechanism with additive/cooperative interaction potential that is mediated by the opening of junctions of adjacent endothelial cells (via para-cellular) or through the cell body (transcellular) [25,26].

Cell adhesion molecules are glycoproteins expressed on the cell surface that mediate contact between two cells or between cells and the endothelium. Three families of adhesion molecules have been identified: (1) selectins, which are predominantly responsible for the initial contact of leukocytes with the endothelial vasculature in the leukocyte recruitment and “rolling” stages [27]; (2) integrins, which are composed of the α subunit, also known as CD11, and β subunit and mediate the firm adhesion of leukocytes to the endothelium, especially integrins of the β2 or CD18 family [28]; and (3) CAMs (cell adhesion molecules), proteins belonging to the immunoglobulin superfamily that are involved in adhesion and migration between leukocytes and endothelial cells [27,28].

Few studies have examined the participation of different domains of metalloproteases in inflammatory reactions, and even fewer have assessed their roles in modulating the expression of adhesion molecules during the process of leukocyte recruitment and cell migration after exposure to these toxins. An understanding of the mechanisms that contribute to the inflammatory response caused by *Bothrops* envenomation may help resolve the local reaction observed. Thus, this study investigated the effects of different SVMP domains on modulating the expression of adhesion molecules on leukocytes and the microvasculature of mice exposed to three different toxins isolated from *Bothrops* venoms: Jar and Jar-C, which are a PIII-SVMP and disintegrin-like protein isolated from *Bj*V, respectively, and BnP1, a PI-SVMP with weak hemorrhagic action isolated from *Bn*V.

## 2. Results

### 2.1. Expression of the ICAM-1 and PECAM-1 Proteins in the Cremaster Muscle

**Figure 1:**
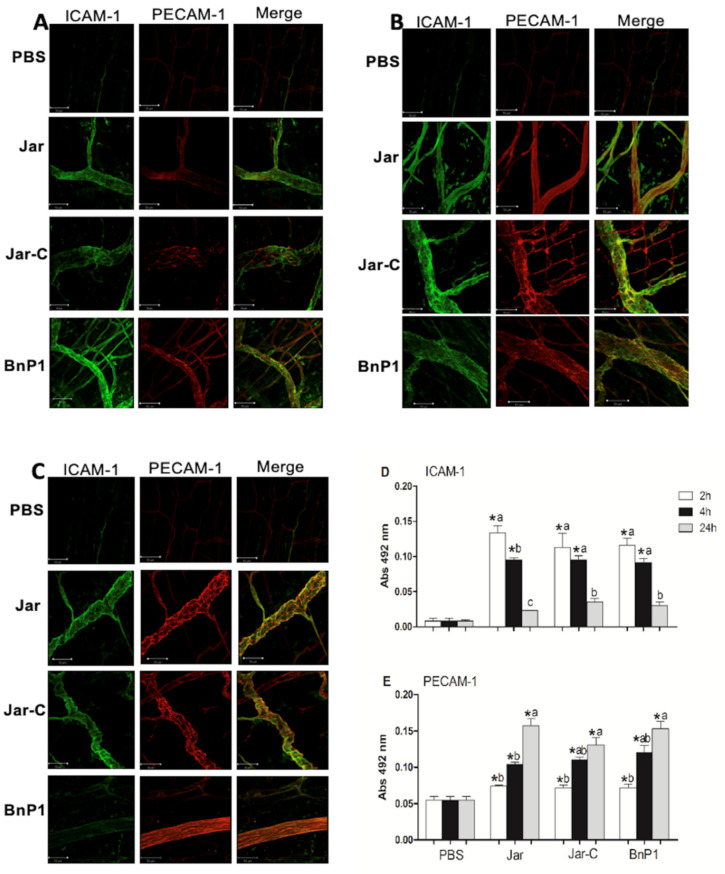
Expression of ICAM-1 and PECAM-1 in the cremaster muscle at different time points. The panels present photomicrographs of immunofluorescence labeling analyzed using confocal microscopy at 2 h (**A**), 4 h (**B**) and 24 h (**C**) after the subcutaneous injection of PBS, Jar, Jar-C or BnP1 (0.5 μg/100 μL) in the mouse scrotum. Bar, 50 µm. Quantification of the protein levels of ICAM-1 (**D**) or PECAM-1 (**E**) in homogenates of cremaster muscle determined using ELISA. Absorbance was measured at 492 nm. The results are presented as the means ± S.E.M. (*n* = 5). * *p* < 0.05 compared to the PBS group at any time point. Different letters (a, b, c) in the same group represent significant differences (* *p* < 0.05).

The microcirculation of the cremaster muscle in animals injected with the toxins Jar, Jar-C, and BnP1 presented positive labeling for both adhesion molecules compared to the microcirculation of the control group. When the levels of these proteins were quantified using ELISA, these three toxins induced a similar pattern of protein expression based on the level of fluorescence and time course of appearance (Figure 1A–E).

Toxins induced an increase ICAM-1 expression in the early stages (2 h and 4 h) compared with the control group (Figure 1A,B); however, at 24 h postinjection, the group that received BnP1 did not exhibit a morphological difference from the control group (Figure 1C). Quantification using the immunoassay confirmed the increase in the expression of ICAM-1 in groups injected with the toxins 2 and 4 h after the injection compared to the control group. A statistically significant difference was observed in animals injected with Jar between 2 and 4 h, and this pattern was exclusively induced by Jar (Figure 1D). Twenty-four hours after the injection of those three toxins, the concentration of ICAM-1 returned to a value similar to its initial level and did not differ from the control group (Figure 1D).

PECAM-1 expression in the microvasculature of cremaster muscle was significantly increased 4 h and 24 h after the injection of the three toxins when compared to the control group, as evidenced by the results of immunofluorescence assays (Figure 1B,C). The quantification of PECAM-1 levels using ELISA revealed constitutive expression in the control group, and an evident increase in the expression of this molecule was induced by the three toxins over time. This difference was greater at 24 h after toxin injection (Figure 1E). Notably, no nonspecific immunofluorescence labeling for the primary antibody was observed, since staining was also conducted using samples from in naive animals (data not shown).

### 2.2. Expression of mRNAs Encoding the Adhesion Molecules ICAM-1 and PECAM-1 in the Cremaster Muscle of Mice

**Figure 2:**
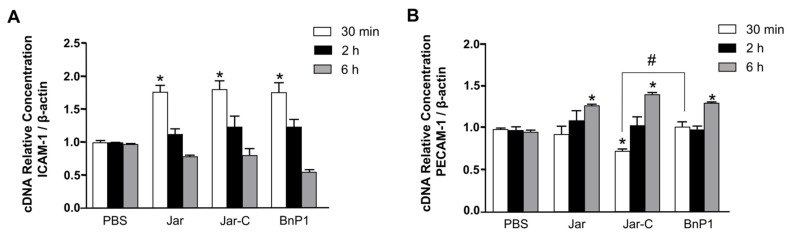
Expression of mRNAs encoding the adhesion molecules ICAM-1 (**A**) and PECAM-1 (**B**) in cremaster muscle. Jar, Jar-C, BnP1 (0.5 μg/100 μL) or PBS (100 µL) was injected, and the muscle was isolated for total RNA extraction. Gene expression was quantified using real-time PCR. Graph bars show the relative expression of each mRNA compared with the PBS group after normalization to the housekeeping gene β-actin. The results are presented as the means ± S.E.M. of three independent experiments (*n* = 5) analyzed in triplicate. * *p* < 0.05 compared to samples treated with the control, # *p* < 0.05 compared to the BnP1 group.

The expression of the mRNAs encoding ICAM-1 and PECAM-1 present on the vascular endothelium of the cremaster muscle of mice was evaluated in samples obtained at 30 min, 2, and 6 h after the injection of Jar, Jar-C or BnP1 using real-time PCR and compared with the nontreated control group (PBS). According to our results, the three toxins increased the expression of the mRNA encoding the cell adhesion molecule ICAM-1 at 30 min after toxin injection (Figure 2A).

The PECAM-1 mRNA expression pattern differed from that observed for ICAM-1. PECAM-1 was expressed later (6 h) (Figure 2B). At the early time points of 30 min and 2 h, the toxin-injected groups showed no significant differences from the control group. The profile induced by Jar-C showed a slight but significant difference compared to the PBS and BnP1 groups, as PECAM-1 expression decreased in the first 30 min (Figure 2B).

### 2.3. Expression of CD11a and CD11b on the Surface of Leukocytes Present in the Peritoneal Exudates from Mice Injected with Different Toxins

**Figure 3:**
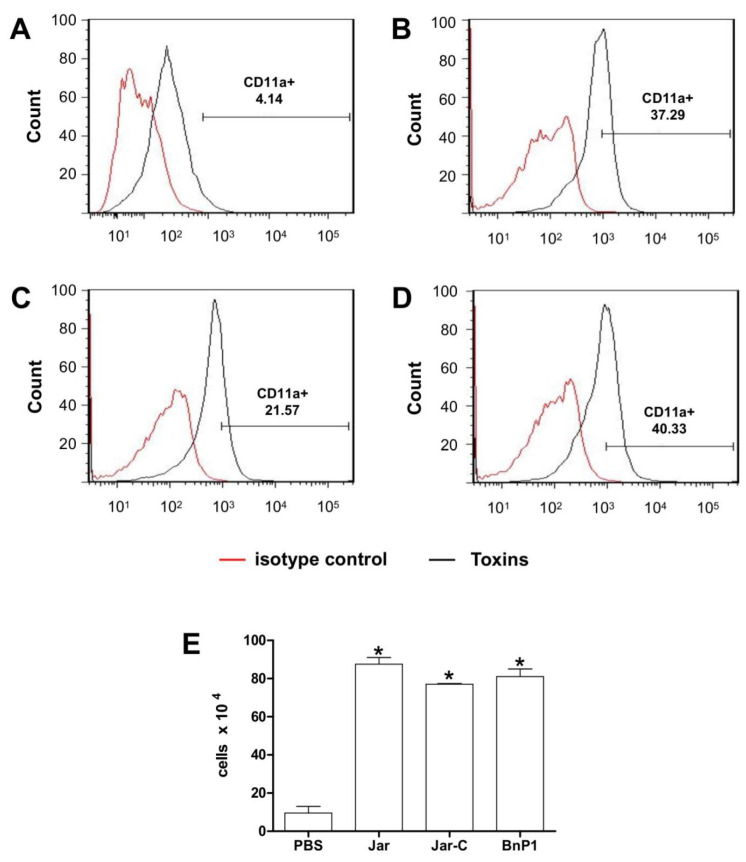
Expression of CD11a on the surface of leukocytes present in the peritoneal exudate of mice injected with different toxins. Peritoneal exudate cell suspensions were obtained within 4 h after the injection of PBS (**A**), Jar (**B**), Jar-C (**C**) or BnP1 (**D**) (2 μg/300 μL). Cells were incubated with anti-CD11a-FITC antibodies or isotype control-FITC. All incubations with anti-CD11a-FITC were performed on duplicate samples and analyzed using flow cytometry. Histograms are representative of one experiment. The bar graph shows the average numbers of CD11a-positive cells in each experimental group ± SD from three independent experiments (**E**). * *p* < 0.05 compared to the PBS group.

**Figure 4:**
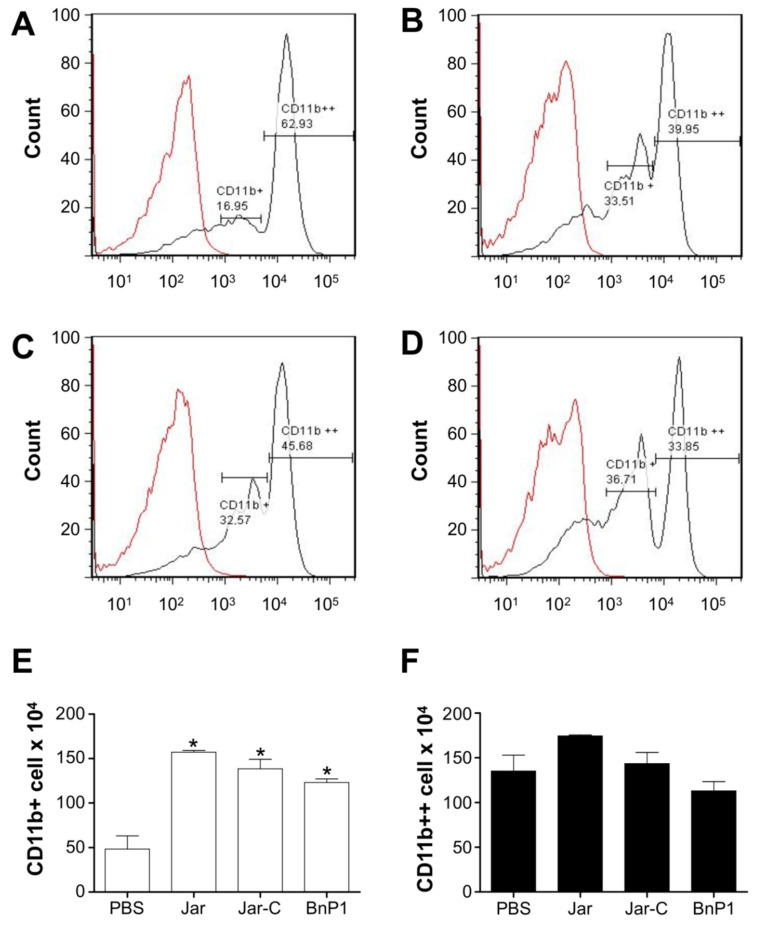
Expression of CD11b on the surface of leukocytes present in the peritoneal exudate of mice injected with different toxins. Peritoneal exudate cell suspensions were obtained within 4 h after the injection of PBS (**A**), Jar (**B**), Jar-C (**C**) or BnP1 (**D**) (2 μg/300 μL). Cells were incubated with anti-CD11b-FITC antibodies or isotype control-FITC. All incubations with anti-CD11b-FITC were performed on duplicate samples and analyzed using flow cytometry. Histograms are representative of one experiment. The bar graph shows the average numbers of positively stained cells designated as CD11b+ (**E**) or CD11b++ (**F**) in each experimental group ± SD from three independent experiments. * *p* < 0.05 compared to the PBS group.

The expression of the CD11a and CD11b adhesion molecules on the surface of leukocytes present in the mouse peritoneal exudates was investigated using flow cytometry 4 h after the injection of Jar, Jar-C or BnP1 and compared to the control group (Figure 3 and Figure 4). Leukocytes from the PBS-treated group showed basal expression of CD11a (Figure 3A,E), and the number of cells expressing this molecule increased in the groups of mice injected with the three toxins (Figure 3B–E).

The expression of CD11b was not detected in peritoneal exudate cells from the control group. However, two other cell populations showed differences in the expression of the CD11b molecule, i.e., one population exhibited intermediate expression of CD11b and was designated CD11b+ cells, and the other exhibited high expression of this molecule and was designated CD11b++ cells (Figure 4A,E,F). In groups injected with the toxins, an increase in the CD11b+ population was observed compared to that observed in the exudate from the group injected with PBS (Figure 4A–E), but no differences in the CD11b++ population were observed (Figure 4F).

## 3. Discussion

As shown in our study, three SVMP toxins, Jar, Jar-C, and BnP1, isolated from *Bothrops* venoms, induce the expression of adhesion molecules on the microvasculature of murine cremaster muscle. Each of these toxins shows essential differences in the compositions of their structural domains. In general, PIII SVMPs (Jar) are more hemorrhagic than PI SVMPs (BnP1), although both degrade extracellular matrix molecules [29,30]. The stronger hemorrhagic activity of PIII SVMPs has been attributed to the presence of a disintegrin-like domain adjacent to the catalytic domain. This disintegrin domain favors the anchoring of the molecule to the basement membrane, amplifying the hemorrhagic potential [30]. Some SVMPs of *Bothrops* venoms degrade components of the basement membrane, increasing vascular permeability, and induce the expression of adhesion molecules [30,31]. Although Jar-C does not contain the catalytic domain, the ECD-disintegrin and cysteine-rich domains have also been shown to be essential for the inflammatory function of these toxins [9,16].

Unlike the hemorrhagic action of the SVMPs, the toxins Jar, Jar-C, and BnP1 exerted similar effects on inducing the expression of adhesion molecules responsible for changes in the leukocyte-endothelium interaction, regardless of the composition of their domains. Our results reveal the responses of endothelial and leukocyte cells to the presence of toxins through the expression of the key molecules ICAM-1, CD11a, and CD11b in the early stages of cellular migration and the expression of PECAM-1 in the late stages studied (Figure 1, Figure 2, Figure 3 and Figure 4).

Leukocyte recruitment is regulated by factors that alter microvessels to promote firm adhesion and cell migration to the site of infection or tissue injury, inducing inflammation and subsequent tissue repair [25]. A cascade of events occurs to achieve this effect, including endothelial cell activation, the release of inflammatory mediators and the expression of adhesion molecules. These processes are often amplified by pathogen-associated molecular patterns (PAMPs) [32], damage-associated molecular patterns (DAMPs) [33] or venom-associated molecular patterns (VAMPs) [34] that function as alarmins stimulating the innate immune response alone or through interactions with cytokines and chemicals.

*Bothrops* venoms induce inflammatory responses and activate signaling pathways that culminate in the transcription of inflammatory genes such as cytokines and eicosanoids [35,36], inducing endothelial activation to promote capture, rolling, firm adhesion and cell migration [37,38].

Previous studies by our group have identified the critical role for SVMPs in the proinflammatory cytokine action induced by *Bothrops jararaca* and *Philodryas patagoniensis* venoms; both venoms contain a large amount of metalloproteases [8,39]. Additionally, we recently reported that Jar, Jar-C, and BnP1 induce cell adhesion and migration in postcapillary venules in the cremaster muscle of mice, as observed using intravital microscopy [9]. These data corroborate the increase in the expression of adhesion molecules observed in the present study.

Other PIII SMVPs, namely HF3 isolated from *Bothrops jararaca* venom and patagonfibrase isolated from the Dipsadidae snake *Philodryas patagoniensis*, also induce changes in leukocyte-endothelial interactions [39,40].

The recruitment of leukocytes to the injured tissues is a characteristic event of the inflammatory response, and the leukocyte-endothelium interaction is promoted by the expression of cell adhesion glycoproteins on the surface of leukocytes and endothelial cells. This event occurs mainly when integrins on leukocytes bind to adhesion molecules on the endothelium [23,24,25,41].

Increases in the expression of ICAM-2, PECAM-1, and Jam-A have been described in the literature after the injection of TNF-α and IL-1β, and the interaction between these adhesion molecules facilitates the migration of leukocytes to extravascular tissue [25,41]. Notably, the proinflammatory role of Jar might be attributed to its ability to process the tumor necrosis factor (TNF-α) precursor [7], and it is still capable of inducing the mRNA expression of proinflammatory cytokines such as TNF-α, IL-6, and IL-1β [42]. Additionally, once the leukocyte adheres to ICAM-1, this binding increases its avidity through interactions with integrins, facilitating the transmigration process on the leukocyte surface [43]. This observation might explain the increased expression of ICAM-1, CD11a and CD11b observed in the first hours evaluated after the injection of the different toxins (Figure 1, Figure 2A, Figure 3 and Figure 4).

In general, leukocytes are recruited during an inflammatory insult, and this mobility is essential for the defense of the organism. Neutrophils are more frequently observed and are quickly recruited and activated [41], although other circulating leukocytes also participate in this process, depending on the inflammatory stimulus. Based on our results, both LFA-1 (CD11a/CD18) and Mac-1 (CD11b/CD18) are expressed after the injection of toxins, suggesting that both adhesion molecules participate in this leukocyte-endothelium interaction. However, the differences between these molecules should be highlighted. Both LFA-1 and MAC-1 are members of the β2 integrin family but are expressed in different cell types. MAC-1 is present on neutrophils and monocytes [25,42], identifying the two types of cell populations present in the peritoneal exudate from mice 4 h after the injection of the different toxins (Figure 4), whereas LFA-1 is expressed on all effector leukocytes (Figure 3).

Phillipson et al. [44] described that the adherence of neutrophils to the activated endothelium is mainly mediated by LFA-1, but intraluminal crawling, which follows adherence and spreading, depends on MAC-1. Sumagin et al. [45] observed an increase in CD11b expression in monocytes and a decrease in this expression in neutrophils in postcapillary venules of the cremaster muscle of mice without inflammatory stimulation. Some authors have also shown that the MAC-1 blockade prevents leukocyte recruitment [46,47]. The balance and regulation of this signaling via integrins contribute to leukocyte transmigration guided by endothelial cells.

PECAM-1 is the central adhesion molecule related to cellular transmigration. This protein is expressed at high levels in endothelial cells. It accumulates in intercellular contacts and is associated with the junctional regulation of endothelial complexes such as β-catenin, VE-cadherin, and adherent endothelial junctions, signaling remodeling of the cytoskeleton during the transmigration process [44,48]. We observed a significant increase in the expression of the PECAM-1 mRNA (Figure 2B) and protein (Figure 1E) in the cremaster muscle of mice injected with all toxins at the later time points compared with the ICAM-1 molecule that is responsible for firm adhesion, which was expressed in the first studied periods (Figure 1D and Figure 2A).

These molecules have also been shown to play important roles in other studies examining *Bothrops* venoms. L-selectin, ICAM-1, and PECAM-1 molecules participate in the leukocyte recruitment induced by *Bothrops asper* venom, or BaP1, a P1 SVMP [31,35]. This inflammatory effect was related to the actions of inflammatory cytokines and leukotrienes released by the venom that act on endothelial cells and leukocytes [49].

The results obtained in the present study confirm the importance of metalloproteases in inducing the local inflammatory response observed in previous studies [49,50,51], particularly in microcirculatory changes [8,9]. Nevertheless, our results reveal the initial participation of the molecules ICAM-1, CD11a, and CD11b and the late expression of PECAM-1. This expression follows the pattern of cell adhesion and migration in vivo and is compatible with acute inflammation and consistent with results observed in other studies [9,38]. The participation of other adhesion molecules in the observed changes should not be excluded.

## 4. Conclusions

In the present study, we compared different SVMPs: Jar, a PIII with high hemorrhagic activity, Jar-C, which is devoid of hemorrhagic activity, and BnP1, a PI SVMP with low hemorrhagic activity. Despite the differences in the composition and hemorrhagic actions of these toxins, they similarly modulate the expression of adhesion molecules that promote leukocyte recruitment. In addition, their expression followed a sequential pattern, consistent with the recruitment and adhesion processes that were observed in previous in vivo studies performed using intravital microscopy. Therefore, we suggest a possible mechanism underlying the leukocyte-endothelium interaction after exposure to snake venom toxins. A similar pattern was observed even in other inflammatory pathologies. These data contribute to the understanding of local reactions observed after envenomation induced by *Bothrops* snakebites. The same approach can be used to evaluate if other toxins, such as PLA_2_ and LAAO present in other *Bothrops* venoms, contribute to the inflammatory process observed in this envenoming.

## 5. Materials and Methods

### 5.1. Animals

Male Swiss mice weighing 20–25 g supplied by the housing facility of the Instituto Butantan were used. The animals were maintained for two days in a 12:12 h light: dark cycle and received water and food ad libitum, before experiments. All experimental procedures were conducted according to the ethical parameters proposed by the International Society of Toxinology and the Brazilian College of Experimental Animals and were approved by the Ethical Committee for the Use of Animals of Butantan Institute (n° 466/08).

### 5.2. Toxins

Venoms of *Bothrops jararaca* and *B. neuwiedi* were obtained from snakes housed in captivity at the Butantan Institute Serpentarium (Laboratório de Herpetologia). Jar and Jar-C were isolated from *B. jararaca* snake venom, and BnP1 was isolated from *B. neuwiedi* snake venom, as previously described [13,19,21]. The purity of toxins was tested using 12.5% SDS–PAGE under reducing conditions [52], and all samples were subjected to treatment with Triton X-114 to remove eventual remaining LPS contamination [53]. After Triton X-114 treatment, LPS absence was confirmed by the Limulus Amebocyte Lysate test (LAL, Charles River). Jar and BnP1 used for the LAL assay were previously heated at 70 °C to inactivate the proteolytic activity, and the biological activities of toxins after Triton X-114 treatment were tested by determining the hemorrhagic activity (Jar and BnP1) or by performing a platelet aggregation inhibition assay (Jar-C). All the toxins used in the experiments were LPS-free. The doses of toxins used were based in a previous study [9].

### 5.3. Expression of Adhesion Molecules

#### 5.3.1. Evaluation of Protein Expression in Endothelial Cells of the Cremaster Muscle Using Immunohistochemistry

Adhesion molecules expression was assessed 2, 4, or 24 h after the subcutaneous administration of toxins (0.5 μg/100 μL) or 100 µL of phosphate-buffered saline (PBS, as a negative control) in the scrotum (*n* = 5/group). For the immunohistochemical study, animals were anesthetized and transcardiacally perfused with PBS followed by a fixative solution comprising 4% paraformaldehyde (PFA) dissolved in 0.1 M phosphate buffer (PB, pH 7.4). After perfusion, the cremaster muscle was collected, postfixed with 4% PFA for 6 h, and then transferred to cryoprotectant solution of 30% sucrose in PB for 48 h. The cremaster muscle was incubated with anti-ICAM-1 and anti-PECAM-1 primary antibodies (1:100, Biosciences Product) diluted in 0.3% Triton X-100 for 48 h at room temperature with constant agitation. The tissue was washed with PBS (3 times/10 min) and incubated with the secondary antibody (FITC-conjugated AffiniPure goat anti-mouse IgG and TRICT-conjugated AffiniPure, Jackson ImmunoResearch Laboratories; diluted 1:50 in 0.3% Triton X-100) for 2 h at room temperature. After three washes with PBS, the tissues were mounted on slides with Vectashield (Vector Labs, Burlingame, CA, USA) [30]. The tissues were stored at 4 °C, and the fluorescence was measured using a Zeiss confocal microscope (Zeiss LSM Image Browser program version 4.0.0.157).

#### 5.3.2. Evaluation of Protein Expression in Endothelial Cells of the Cremaster Muscle Using Indirect ELISA Assays

Adhesion molecules expression was also evaluated using indirect ELISA assays [54] after the same treatment described for the immunohistochemical assay. The cremaster muscle was removed, placed in 200 µL of 1 M Trisma solution (pH 7.5) containing protease inhibitors (EDTA 1 mM, PMSF 2 mM and aprotinin 0.3 µM), and disrupted with an ultrasonic homogenizer. The homogenate was centrifuged at 12,000× *g* for 20 min at 4 °C, and the protein present in the supernatant was quantified using the Bradford method. ELISA microplate wells were coated overnight at 4 °C with 50 µg of protein diluted in 100 mM carbonate-bicarbonate buffer (pH 9.6). The plates were washed three times with PBS-Tween (0.05%) and blocked with 1% bovine serum albumin (BSA) for 1 h at 37 °C. The plates were washed again and incubated overnight at 4 °C with anti-ICAM-1 and anti-PECAM-1 antibodies (1:500, Biosciences Product). Then, after a new series of three washes, the plates were incubated for 2 h at 37 °C with anti-IgG conjugated with peroxidase (1:2000 —Jackson ImmunoResearch Laboratories). The antibodies were diluted in 1% BSA/PBS-Tween 0.05%. The reaction was visualized using 0.7 mg of OPD (o-phenylenediamine) diluted in 100 mM phosphate-citrate buffer (pH 5.5) containing 0.05% H_2_O_2_, stopped after 10 min by adding 50 µL/well of 8 N H_2_SO_4_, and quantified by reading the absorbance at 492 nm using an ELISA plate reader (Multiskan EX Thermo Scientific). All ELISA experiments were performed in triplicate in three independent experiments.

### 5.4. Real-Time PCR Analysis of Gene Expression in Endothelial Cells of the Cremaster Muscle

The expression of adhesion molecule transcripts was measured 30 min, 2 h, and 6 h after the subcutaneous administration of toxins (0.5 μg/100 μL) or PBS (100 mL) as a negative control into the scrotum. Total RNA was extracted from the dissected cremaster muscle in TRIzol solution (Invitrogen) using an ultrasonic homogenizer. Five micrograms of total RNA were reverse transcribed into cDNAs using Superscript III RT (Invitrogen). Quantitative real-time PCR was performed in a Line Gene K Thermal Cycler (Hangzhou Bioer Technology Co., Hangzhou, China) using a Platinum Syber Green qPCR Supermix-UDG kit (Invitrogen) with the following thermal cycling protocol: 15 min at 95 °C followed by 40 cycles of 15 s at 95 °C, 30 s at 58 °C, and 30 s at 72 °C. All procedures were performed according to the manufacturer’s instructions. The primer sequences for mouse β-actin (FW: 5′CCCAGGCATTGCTGACAGG3′; RV: 5′TGGAAGGTGGACAGTGAGGC3′) were designed using sequence alignments obtained from NIH/NCBI based on the published RNA sequence. The primer sequences for mouse ICAM-1 (FW: 5′AAGGAGATCACATTCACGGT3′; RV: 5′GCCTCGGAGACATTAGAGAA3′) and PECAM-1 (FW: 5′AGAGACGGTCTTGTCGCAGTA3′; RV: 5′CGCACACCTGGATCGG3′) were obtained from the literature [55]. The relative mRNA expression level was determined using the 2^−ΔΔ^Ct (cycle threshold) method, and data were normalized to β-actin mRNA expression levels. All real-time PCR experiments were performed in triplicate using samples from three independent animals.

### 5.5. Integrin Expression in Peritoneal Leucocytes

The expression of CD11a and CD11b integrins in leukocytes that migrated to the peritoneal cavity in response to toxin injection was evaluated using flow cytometry. For this experiment, the mice were injected (i.p.) with toxins (2.0 µg/300 µL) or sterile PBS and sacrificed in a CO_2_ chamber 4 h later. Their peritoneal cavities were washed with 5.0 mL of PBS. The peritoneal fluid was collected and centrifuged (1000× *g*/10 min at 4 °C), and the precipitate was resuspended in red blood cell lysis buffer and centrifuged (1000× *g*/10 min at 4 °C). The pellet was resuspended in 1 mL of PBS. The total leukocyte count was determined using an automatic veterinary hematology analyzer (Mindray BC 2800 VET). The peritoneal cells from different animals were pooled to obtain a concentration of 10^6^ cells/mL. Cells were incubated with anti-mouse FcγRII/RIII at a concentration of 1 μg/10^6^ cells (BD System) for 30 min at 4 °C followed by washes with PBS containing 1% serum albumin (BSA) and centrifugation (300× *g*/10 min). The expression of different molecules was analyzed by incubating the cells with 1:200 dilutions of Fluorescein (FITC)-conjugated anti-mouse CD11a and CD11b antibodies for 30 min at 4 °C. All cell suspensions were also incubated with the respective fluorescein-labeled isotype control monoclonal antibodies (e-Bioscience). The cells were washed and resuspended in PBS containing 0.1% paraformaldehyde. The flow cytometry analyses (10^4^ events per data acquisition file) were performed with FACSCalibur using Cell Quest software (Becton Dickinson). All flow cytometry experiments were performed in triplicate in three independent experiments.

### 5.6. Statistical Analysis

The results are presented as the means ± standard errors of the different mice/group for each experiment and were analyzed using one-way ANOVA followed by Tukey’s test. Differences in results were considered statistically significant when *p* < 0.05.

## Data Availability

The data presented in this study are available on request from the corresponding authors.
